# Community-based Kangaroo Mother Care in low-birth-weight infants: a quality improvement study in the urban slums of Kolkata, West Bengal

**DOI:** 10.3389/fped.2026.1754022

**Published:** 2026-03-12

**Authors:** Sweta Halder, Tila Khan, Naresh Kumar Gupta, Rumeli Das, Sreeparna Ghosh Mukherjee

**Affiliations:** Health & Nutrition,Child in Need Institute (CINI), Pailan, West Bengal, India

**Keywords:** breastfeeding, community initiated care, feasibility, infant, kangaroo mother care method, low birth weight, neonate

## Abstract

**Background:**

Kangaroo Mother Care (KMC) is recommended for low-birth-weight (LBW) infants in both facility and community, yet in India, KMC is largely confined to healthcare facilities. Evidence on the feasibility of implementing community-based KMC (c-KMC) in urban slum settings is limited. This quality improvement exploratory study explored the feasibility, safety, and adherence of implementing c-KMC in the urban slums of Kolkata, India.

**Methods:**

A preliminary qualitative assessment explored barriers to c-KMC through in-depth interviews with mothers of LBW infants (*n* = 3) who received facility-initiated KMC, and two focus group discussions with frontline health workers (*n* = 6). From September 1, 2023 to July 31, 2024, KMC was implemented on stable LBW newborns (≤2,200 g; *n* = 218) in community settings. Mothers received counselling, training, and KMC kits. Home visits were conducted on days 7, 14, and 28 after birth or discharge to reinforce KMC and breastfeeding.

**Results:**

Mothers had limited KMC knowledge and received minimal counselling, or follow-up post-discharge. Frontline health workers cited workload, population density, migration, and space constraints as major barriers to c-KMC. Among 218 infants enrolled, only 23.4% received facility-initiated KMC. Average KMC duration was 3.4 h/day at homes across follow-up visits. By day 28, 95% of mothers continued KMC, no KMC related adverse event was noted and 84% practiced exclusive breastfeeding.

**Conclusion:**

c-KMC implementation was feasible and safe in slum settings when supported through structured counselling, home visits, and trained workers. The findings support the operational feasibility of integrating c-KMC within existing home-based newborn care platforms.

## Introduction

The neonatal period, defined as the first twenty-eight days of life, is the most vulnerable period accounting for nearly 2.3 million deaths globally in 2023 (1). In 2024, India contributed approximately 0.4 million neonatal deaths globally (2), with an overall neonatal mortality rate of 17.3 [95% CI: 14.75–20.11] in 2023 (3). Low-birth-weight (LBW), defined as birth weight less than 2,500 g, is a major determinant of neonatal morbidity and mortality, particularly in low-resource settings. South Asia accounts for over 40% of LBW births worldwide (4). In India, 27% of live births are LBW with higher prevalence in the east (33%; 95% CI: 29%–37%) (4, 5). Interestingly, LBW prevalence has been seen to be higher in urban areas (30%; 95% CI: 23%–38%) as compared to the rural (26%; 95% CI: 22%–30%) (5).

Nearly 83% of neonatal deaths in India occur due to LBW complications (6). LBW infants are at increased risk of severe morbidities, infections, and delayed growth and delayed neurodevelopment during the childhood (7, 8). Kangaroo Mother care (KMC), or early, continuous skin-to-skin contact of mother's chest with the baby along with exclusive and frequent breastmilk feeding, is an established method to reduce the mortality among LBW infants (9, 10). KMC supports weight gain, thermoregulation of infant, stimulates breast milk production, promotes exclusive breastfeeding, strengthens mother-infant bonding, and may reduce morbidity (11). A Cochrane review reported that KMC after stabilization reduces mortality in LBW infants by 40% as compared to conventional care (Risk Ratio 0.60, 95% CI: 0.39–0.92) (12).

The World Health Organization (WHO) recommends initiation of Kangaroo Mother Care for fully stable infants weighing 2,000 g or less at birth in health-care facilities (13, 14). In addition, the WHO policy states continuation of KMC at home after discharge. However, the current operational guidelines in India focus predominantly on facility-initiated KMC, where the KMC coverage remains very low, largely limited to tertiary health facilities and medical colleges with sick newborn care units (SNCU) and newborn stabilization units (NBSU) (15, 16).

Community-initiated Kangaroo Mother Care (c-KMC) involves the initiation and continuation of KMC at the household or community level for stable LBW infants soon after birth or discharge. c-KMC is introduced and supported through structured home visits and counselling by trained community health workers, aiming to extend KMC to infants who may miss adequate facility-based care, particularly in settings with high home-birth rates and early discharges. Although, the Home-Based Newborn Care (HBNC) guidelines in India recommend that Accredited Social Health Activists (ASHA) counsel mothers of low birth weights on KMC during routine home visits (17), it is not yet part of a national policy and is not routinely practiced.

A randomized controlled trial from Northern India demonstrated that c-KMC improved neonatal survival by 30% and 6-month infant survival by 25% (18). However, evidence on c-KMC implementation in urban settings—especially in densely populated slums with high LBW prevalence—remains limited. One pilot study from slum areas of western India introduced KMC at homes in 2019 and saw a wide adoption and weight improvement reported in 67% of LBW infants (19). Building on this evidence, the present study was designed not to evaluate clinical impact, but to assess the feasibility, safety, and adherence of implementing community-based KMC in densely populated urban slums of Kolkata. We conducted a situational assessment of the existing facility-initiated KMC services in Kolkata city, in eastern India and identified barriers to implementing c-KMC at the community level. Subsequently, we did a quality improvement implementation of c-KMC within an ongoing maternal and child health program. This study aimed to generate operational insights to inform scalable integration of c-KMC into home-based newborn care services in urban low-resource settings. This was done as part of a large longitudinal, multimodal, community-based maternal and child health and nutrition program of the Child in Need Institute (CINI) that encompassed identification and follow-up of high-risk pregnant women and neonates as a continuum of care along with community engagement and health system support (20).

## Method

### Study design

We conducted a quality improvement study in two phases. In the first phase, we explored the experience of LBW mothers with facility-based KMC, and assessed the knowledge and practice of KMC of honorary health workers (HHW) through focus group discussions. In the second phase, CINI implemented community-based KMC in urban slums to evaluate its feasibility in real world settings as a quality improvement study within the existing maternal and child continuum of care intervention.

### Study site

This study was conducted between June, 2023 and July, 2024, in the urban slums of Kolkata, the capital city of West Bengal, in eastern India. Kolkata, under the jurisdiction of the Kolkata Municipal Corporation, has a population of nearly 4.5 million and the state's highest population density (24,252 km^2^, Census 2011) (21). Nearly-one third of city's residents live in slums- “bustees” often along roads and canals, with inadequate sanitation, poor housing conditions, and limited access to health services, quality education and food (22, 23). These settlements are inhabited largely by migrant labourers and their descendants. Utilization of maternal and newborn health services in these areas remains suboptimal due to overcrowding and stretching of infrastructure and resources, with delayed antenatal registration, low iron and folic acid (IFA) consumption, inadequate screening for anaemia and hypertension, insufficient antenatal counselling, and limited neonatal care interventions and postnatal home visits.

Kolkata comprises 16 boroughs (administrative divisions) that include 144 wards. Our maternal and child health intervention is being implemented in Boroughs 3, 4, 6, 7 and 15. For the c-KMC intervention, we also did purposive sampling of mother-infant dyads from neighbouring boroughs (1, 5, 9 and other wards of borough 3) with comparable socio-economic status to achieve maximum enrolment.

### Qualitative research

To understand the prevailing situation of facility-based KMC being provided in Kolkata medical colleges and the need for a community-based KMC model, a short qualitative exploratory assessment was undertaken randomly with mothers of LBW babies and frontline workers from 9th to 15th June, 2023 prior to start of KMC intervention in the community. Tools for in-depth interviews (IDI) were developed for mothers of LBW babies who had received KMC services in hospitals in last three months for understanding their knowledge, attitude, practice and experience of KMC. Questions focused on socio-demographic background, birth details, and nine questions on their knowledge and care for LBW, details of care and advice received in the facility, post-natal visit of health workers for newborn care, weight monitoring by health system, KMC practice and challenges faced during KMC.

Tools for focus group discussions (FGD) with the government Honorary Health Workers (HHW) or Urban ASHAs were developed to understand the existing health services for LBWs and the barriers of KMC implementation in the community. Questions focused on their training, their awareness about KMC, the care provided to LBWs, follow-up of LBWs, and challenges faced in ensuring community KMC. IDIs and FGDs were conducted by a trained team leader and a peer educator face-to-face in Bengali or Hindi at urban primary health centres for the HHWs and at homes for mothers after informed consent. Interviews were audio recorded and lasted 30–40 min.

### Enrollment and counselling of study participants for community-based KMC

CINI's maternal and child health (MCH) continuum of care intervention involved identification and tracking of high-risk pregnancies by our community mobilizers for ensuring early pregnancy registration, four antenatal care visits, consumption of iron and folic acid consumption, monthly gestational weight gain monitoring, and promoted birth preparedness, including adoption for kangaroo mother care (20). Leveraging our existing field presence in Kolkata, this c-KMC implementation study involved promotion of KMC and exclusive breastfeeding in the community settings.

All stable low birth weight (LBW, <2,500 g) newborns in our MCH cohort were screened for eligibility in their homes by our team comprised of a nutritionist, two nurses and two peer educators. We also received the names of LBWs born from outside cohort through the medical institutions in Kolkata, which were also screened for eligibility in their homes. The inclusion criteria comprised stable newborns weighing between 1,200 and 2,200 g, who tolerated oral feeding, exhibited no respiratory distress, had no clinical signs of illness, and their mothers were willing to provide KMC. The exclusion criteria were newborns with birth weight <1,200 g or >2,200 g, unable to tolerate oral feeding, or having severe respiratory distress, or any other serious diseases that require hospitalization. LBW babies (<2,200 g) meeting the selection criteria were identified and after obtaining informed consent from their mothers or caregivers, the mother-baby dyad were enrolled in the study, irrespective of whether they had received facility-based KMC.

When birth was reported, the intervention team visited the household within 72 h of delivery. Once the eligibility was met, the mother-infant dyads were enrolled. Each mother received counselling on the process and benefits of KMC, exclusive breastfeeding and a KMC kit, consisting of two KMC pouches to facilitate skin-to-skin contact, hand mittens, socks, a cap for the baby, and a *paladai* (a traditional feeding utensil) for feeding expressed breast milk. They were demonstrated the steps of KMC, including KMC positioning with the support of a cloth that females use to cover their heads and body for modesty. In addition, mothers were provided with information, education, and communication (IEC) materials to help them understand and adhere to KMC and exclusive breastfeeding. The IEC materials included ludo games, reminder cards in English and Bengali and posters.

### Follow-up of LBW infants till 28 days of age

After recruitment, nurses and peer educators conducted home visits on day 7, 14, and 28 after birth or hospital discharge, to support the mother and caregivers for KMC practice, in line with the national Home-Based New-born Care (HBNC) guidelines until 28 days of age, and as per the home based KMC manual (17, 24). For VLBWs home visits were scheduled on day 3, 7, 14, and 28. The team supported the mothers in breastfeeding positioning, attachment and continuing exclusive breastfeeding. Efforts were also made for home visits together with the frontline workers of the government viz., Honorary Health Workers (HHWs).

### Capacity building of the honorary health workers and community engagement

In addition to providing support to the community, several capacity-building sessions were conducted for HHWs on the technical components of KMC and the home-visit protocol under HBNC. They were extensively trained to deliver KMC education and hand-holding support to mothers, ensuring sustainability of c-KMC practices for premature and LBW newborns, even after the study ends. Orientation sessions were conducted with community-based women's groups such as Mahila Arogya Samiti (MAS) in two wards which helped identify community volunteers to help promote KMC at the community level.

### Data collection and analysis

#### Qualitative

Interviews and FGD reports were transcribed and translated into English language. Themes and subthemes were identified based on the similarities and patterns after being unanimously agreed upon by the study team.

#### Quantitative

On each home visit, the team documented information on the duration of skin-to-skin contact, breastfeeding practices (exclusive breastfeeding and continued breastfeeding practices) in the preceding 24-h, illness history, measured weights thrice using digital counter infant weighing scales (BY80; Beurer, Hollywood, FL, USA; 5 g accuracy) and the number of visits made by the government HHWs for all the study neonates in the neonatal period. Danger signs were explained and the information on skin-to-skin contact was ascertained for the preceding 24 h (number of hours per day).

The study team composed of a team leader, two nurses and two peer educators underwent intensive formal training at King Edward Memorial (KEM) Hospital, Mumbai, on KMC procedure and implementation. Study was monitored and supervised by a program manager and program head. Data was captured on hard copies and uploaded on Microsoft Excel. Binary data was expressed as proportions and continuous as medians and interquartile range (IQR). All data was analysed by STATA software version 15.0 and *p*-values <0.05 were considered significant.

### Data analysis

Community-based KMC exposure was defined as caregiver-reported skin-to-skin contact between mother and infant in the preceding 24 h, assessed during scheduled follow-up visits on days 7, 14, and 28. Due to the community-based implementation context, continuous daily verification of KMC practice was not feasible. Reported duration was categorized descriptively (0–3 h, 4 h, >4 h) and was not treated as a binary exposure or dose threshold.

Missing data across visits and variables resulted from delayed hospital discharge, temporary or permanent migration of families, and unavailability of participants during scheduled home visits. When in-person follow-up was not possible, data on breastfeeding practices, reported KMC duration, and infant weight (if measured at nearby health facilities) were collected telephonically. Analyses were conducted longitudinally among enrolled mother–infant dyads wherever data were available; however, not all variables were available for all participants at all time points. Denominators for each variable and visit are explicitly reported in tables and figures.

## Results

### Experience of facility-initiated KMC of LBW mothers

Three mothers of LBWs from different Kolkata slums (wards 32, 56, 67) who received facility-initiated KMC participated were invited to participate in exploratory in-depth interviews. All were secondary school educated homemakers, from nuclear families, with deliveries occuring at tertiary hospitals. Two had twin births. Although informed that their babies were underweight, none received counselling on the consequences and care requirements for LBW infants. Consistent monitoring for special newborn care was limited to those admitted to the sick newborn care unit (SNCU). While posters and IEC materials on KMC were available in hospitals, practical demonstrations and guidance were lacking. No post-discharge follow-up by HHWs was reported, though two mothers independently revisited facilities for baby weight checks.KMC was first introduced at the facility, but most mothers initially lacked understanding about its procedure, duration, and benefits.

The major reported challenges that the mothers faced during KMC were back pain, discomfort during hot weather, limited support from home as families were nuclear, twin care burden, and lack of rest time.

### Knowledge and experience of honorary health workers

Two exploratory triad discussions were conducted with three HHWs each from wards 46 and 54 having over three years of work experience. They highlighted workforce shortages and high population density in Kolkata- only three HHWs served a population of 12,823 in ward 46-restricting regular household visits for health monitoring. They prioritized outreach to only vulnerable populations at most need or risk. While undergone multiple training programs organized by the Government on antenatal care, high-risk pregnancies, postnatal care, care of lactating mothers, home-based newborn care and COVID-19, none had received formal KMC training highlighting training gaps on KMC practices. One mentioned that she received IEC materials and booklets.

Knowledge levels varied: one was unaware of KMC, another knew only the term but had limited technical understanding of KMC, and the third was unaware of the procedure or objectives. Home visits for LBW follow-up were irregular usually limited to emergencies. They reported not receiving lists of LBW or high-risk infants from facilities, and KMC was rarely practiced in the community. When asked about the challenges they may face in implementing KMC in the community, they highlighted migration, frequent relocation, and space constraints at home.

### KMC intervention: birth details

Between September 1, 2023 and July 31, 2024, around 636 LBW (<2,500 g) newborns were identified, of which 218 mother-infant dyads met the selection criteria and enrolled in the study as described in Table 1. Gestational and obstetric details were unavailable for all. All births occurred at a health facility. The median birth weight was 1980 (IQR: 1,796–2,100) g. Ten (4.6%) were very low birth weight VLBW (<1,500 g) at birth, while none was extreme LBW (<1,000 g). Among the enrolled, 112/218 (51.4%) were females. The majority of families were Hindu (55.5%) followed by Muslims (43.6%). One had a congenital heart disease and one had congenital heart defect. 51% were residents of Kolkata and the rest had relatives in Kolkata and had come for deliveries. Facility-based KMC was initiated in only 23.4% (51/218). All infants were initiated c-KMC irrespective of whether they had received KMC at facility. Ten were twin pairs, where both the infants received KMC. The median duration of hospital stay was 6 (IQR: 5–8) days post-birth, while it was longer for VLBW- 19.5 (13–22) days. Around one-third 71/218 (32.6%) stayed more than 7 days.

**Table 1:** Demographic and birth characteristics of low-birth-weight infants enrolled in the study.

Characteristic	Details	*n* (%)
*N*		218
Sex	Male	106 (48.6%)
	Female	112 (51.4%)
Religion	Hindu	121 (55.5%)
	Muslim	95 (43.6%)
	Other	2 (0.92%)
Permanent Residence	Kolkata	112 (51.4%)
	Outside Kolkata	106 (48.6%)
Birth weight	[median, IQR] g	1,980 [1,796, 2,100]
Birth weight categories	<1,000 g	0
	1,000–1,499 g	10 (4.6%)
	1,500–1,999g	102 (46.8%)
	2,000–2,199 g	106 (48.6%)
KMC initiated in facility	Yes	51 (23.4%)
Hospitalization after birth, days	[median, IQR]	6 [5–8]

### Weight gain and exclusive breastfeeding during the neonatal period

**Figure 1:**
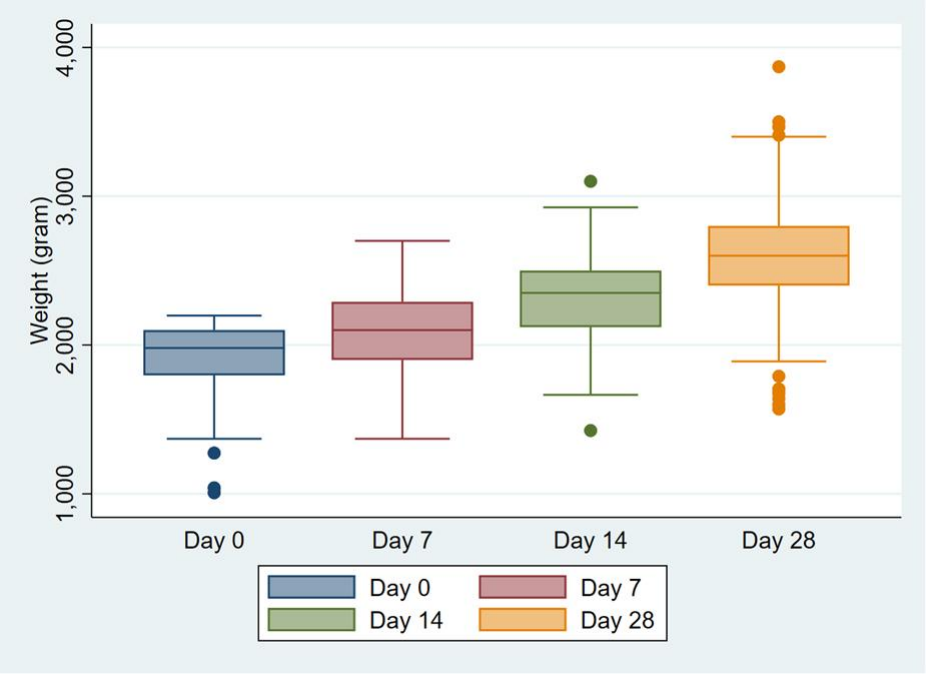
Weights of low-birth-weight infants at birth, day 7, 14 and 28. Weights are presented as box plots where box represents the interquartile range (IQR, middle 50%), with a central line marking the median, whiskers show variability and dots indicate outliers.

The changes in weights are described in Table 2 and Figure 1. By day 7, the majority (196/212, 92.45%) of neonates were exclusively breastfed. Among those admitted for more than seven days after birth, 89% were exclusively breastfeeding exclusively by day 7. The median weight was 2,100 g (IQR: 1,900–2,290), 92% were doing KMC, and the median duration of skin-to-skin contact was 3 h per day (IQR: 2–4). At day 7, 0–3 h of KMC was practiced by 118 mothers; 44 reported 4 h and 39 reported >4 h. Infant weights increased from birth to day 7, consistent with expected postnatal growth trajectories at an average weight gain of 22 g per day. The majority (206/212; 97.2%) were healthy showing no signs of illness, although a few had minor illnesses such as jaundice, cough, skin rash.

**Table 2:** Adherence to community-based kangaroo mother care, duration of practice, exclusive breastfeeding, and infant weight based on 24-hour recall at days 7, 14, and 28.

Indicators	Day 7	Day 14	Day 28
Exclusive breastfeeding (n/N, %)	196/212 (92.45)	166/202 (82.2)	179/214 (83.6)
Weight, grams (median, IQR), N*	2,100 g (IQR: 1,900–2,290) (*N* = 207)	2,350 g (IQR: 2,120–2,500) (*N* = 193)	2,600 g (IQR: 2,400–2,800) (*N* = 206)
Adherence to Kangaroo Mother Care (*n*/*N*, %)**	185/200 (92)	183/190 (96)	194/204 (95)
KMC Duration per day,** hours, median (IQR)	3 (2–4)	6 (5–7)	4 (2–4.5)
0–3 h of KMC per day, (*n*/*N*, %)	118/200 (59)	100/190 (52.6)	92/204 (45)
4 h of KMC per day, (*n*/*N*, %)	44/200 (22)	50/190 (26.3)	62/204 (30.4)
>4 h of KMC per day, (*n*/*N*, %)	39/200 (19.5)	40/190 (21)	51/204 (25)
Minor illness (*n*/*N*, %)	6/212 (2.8%)	8/201 (3.98%)	12/214 (5.6%)
Hospitalization history (*n*/*N*, %)	0/212 (0%)	0/201 (0%)	0/214 (0%)
Mortality (*n*/*N*, %)	0/212 (0%)	0/201 (0%)	0/214 (0%)

* Infant weights were measured by the community mobilizers on home visits conducted on days 7, 14, and 28 or were measured at subcentres for those who migrated. KMC adherence (continuation of KMC practice) and duration is based on maternal self-report for the preceding 24 h at follow-up visits. Data reports practices and infant weight indicators are not intended to infer causal effect.

** IQR, interquartile range.

By day 14, 96% of mothers continued KMC and the duration of skin-to-skin contact increased to 6 h per day (IQR: 5–7). At day 14, 0–3 h of KMC was practiced by 100 mothers; 50 reported 4 h and 40 reported >4 h. Out of 16 infants who did not get KMC at day 7, 15 were getting KMC by day 14, except one whose mother was hospitalized. The median weight increased to 2,350 g (IQR: 2,120–2,500). The majority continued exclusive breastfeeding (166/202, 82.2%) and were healthy 193/201 (96%). Eight were sick from fever, cough and cold, redness of eye, constipation or jaundice.

By Day 28, almost 95% of mothers continued KMC, though the duration of skin-to-skin contact fell to 4 h per day (IQR: 2–4.5). At day 28, 0–3 h of KMC was practiced by 92 mothers; 62 reported 4 h and 51 reported >4 h. The median weight further increased to weight 2,600 g (IQR: 2,400–2,800). Exclusive breastfeeding was maintained in 84%. Nearly two-third, 128 (62.7%) weighed >2,500 g. Sickness was reported in 12/214 (5.6%). No serious safety concern occurred due to KMC in all the study participants and no hospitalization or mortality was recorded during the neonatal period.

Home visit record of HHWs suggested limited HBNC service delivery in the study area. By day 7, only seventy-three households received one visit and five received two visits. By day 14, 73 visits were conducted, and by day 28, 82 visits were recorded.

## Discussion

Despite the well-established benefits of KMC, its implementation in low-resource settings remains suboptimal. This quality improvement study demonstrates that community-based KMC can be implemented safely and with high reported adherence in densely populated urban slum settings when supported through structured counselling, home visits, and trained frontline workers. The findings primarily provide operational insights into feasibility, acceptability, and implementation challenges rather than evidence of clinical effectiveness.

Although the present study lacked a control group, similar community-based implementation studies in India have shown that single-arm interventions can yield meaningful insights into program feasibility and behaviour change (25). A community-based pilot from Odisha, Gujarat and Maharashtra across rural, urban and rural tribal population, found all mothers continued KMC once initiated, although duration varied across the states (25).

Unlike facility-initiated KMC, where at least 8 h of daily KMC is recommended (26), the home-based KMC is often lower due to practical reasons. In our study, the median duration increased to 6 h per day by day 14 and gradually declined to 4 h per day. This decline coincided with commonly reported challenges in continuing KMC in slum settings, including limited family support in nuclear families, limited participation of family members in KMC provision, household responsibilities and privacy constraints in small homes. Similar barriers have been reported in tribal areas of Maharashtra, as well as in Bangladesh and Ghana where household demands limited mothers' ability to maintain prolonged KMC (25, 27–29). The duration of KMC is a challenge even in health facilities with support of nurses where efforts are continuously made to increase the duration of KMC. In our study, we observed 4–6 h of c-KMC that is comparable to findings from a tertiary health facility in Western India (30).

The benefits of early, facility initiated KMC particularly before clinical stabilization, are well established, including improved weight gain, exclusive breastfeeding rates and the reductions in morbidity and mortality among LBW infants (12). In addition, large randomized controlled trials from northern India demonstrated the effectiveness of c-KMC in improving neonatal and six-month infant survival by 30% and 25%, respectively (18), and in enhancing breastfeeding performance and maternal satisfaction (31). The present study was not designed to replicate or evaluate these effects. Instead, it complements existing evidence by highlighting practical considerations for implementing c-KMC in urban slums, including workforce constraints, migration, household space limitations, and follow-up challenges. No major illnesses, hospitalizations, and deaths were observed during the study follow-up period.

Our study highlighted training gaps of government HHWs on KMC and limited routine home visits of HHWs under the HBNC program, which was also found when we did focus group discussions with HHWs underscoring low awareness, workforce shortages and high population density as barriers. The key strength of this implementation study was the training of HHWs in supporting KMC at home, to ensure the continuity beyond the study period. The national guidelines also emphasize continuation of KMC at home linked to home-based care and follow up of the infant by ASHA workers (17). Our results suggest that structured home-based support and counselling by community health workers are feasible and acceptable components of c-KMC implementation in low-resource settings. At the same time efforts are needed to increase the HHWs and their training on KMC for improved service delivery in urban settings.

Prior studies have documented the psychosocial benefits, including reduced maternal stress and depression (32), and equitable impact across socioeconomic groups (33). Our study adds to the growing evidence by demonstrating the operational feasibility of c-KMC in densely populated urban environments, where facility-based KMC coverage remains limited due to high-birth rates and HBNC services are inadequate. These findings may support c-KMC operational feasibility within ongoing maternal and new born health programs in similar urban contexts.

Implementation studies often cannot include a contemporaneous control group due to ethical concerns and operational constraints, as was the case in this study (25). Nonetheless, this non-randomized quality improvement study generated practical insights from the urban slum settings, before implementation at a larger scale, particularly on planning, training frontline workers, human resource and financial support needed and frontline worker competencies in supporting KMC and breastfeeding at home. It also highlighted the need to conduct regular home visits, perform anthropometric measurements and the social behavioural and communication change strategies to mobilize mothers and their families. Since the timing of home visits aligned with the standard HBNC guidelines, the integration of c-KMC into HBNC schedules appeared operationally feasible, requiring no additional visits.

The study has several limitations. The study design does not permit causal inference, and observed clinical patterns cannot be attributed to KMC exposure. Further, the lack of a control group limits us to evaluate the effectiveness of c-KMC on weight gain, breastfeeding and neonatal mortality. The small sample size may not be representative of LBW infants in Kolkata, which restricts us to generalize conclusions. Growth assessment was based only on weight due to funding constraints, preventing a comprehensive evaluation of growth trajectory. Measurement of length was not feasible because of space constraints and uneven surfaces in slum settings. Follow-up ended at day 28 due to which we could not assess KMC's long-term effect on child's growth, cognitive and neurodevelopment outcomes. The use of convenience sampling method may have introduced selection bias and further limits the generalizability of the results to other urban slum contexts in India. In addition, reliance on self-reported data on the duration of skin-to-skin contact and breastfeeding may have introduced recall and social desirability bias, potentially affecting the accuracy of reported duration and adherence to KMC practice at home. The collection of KMC practice data on three time points does not confirm the uninterrupted daily KMC practice. Attrition occurred as some mothers returned to their primary homes or the families relocated during the follow-up period, resulting in a 5% loss of mother-infant dyads, which may have influenced the completeness and precision of follow-up data. Frequent migration practices in slum areas remains an important challenge for follow-up and continuity of care and may affect the broader applicability of the results. Moreover, migration of people from other states also brings cultural and language diversity, that may influence KMC education, acceptance and uptake. Lastly, we could not assess the gestational details, delivery details and the experiences of study families and challenges in continuing KMC at home.

Future large-scale studies utilizing randomized controlled designs with appropriate controls are needed to strengthen causal inference of the impact of KMC on weight gain, breastfeeding and mortality. The use of structured mechanisms to record the duration of skin-to-skin contact and breastfeeding would further enhance the robustness of the findings. Longer follow-up periods are needed to evaluate the long-term outcomes of community-based KMC programs on child growth and development.

Overall, this exploratory quality-improvement study presents the implementation feasibility of integrating KMC within HBNC programs in resource-constrained urban slums settings. The study addresses an important evidence gap, as most KMC data originate from facility-based or rural settings. While c-KMC was well adopted, the findings require validation in larger samples and across diverse urban slum contexts. The results also suggest the need of increasing workforce in urban settings to fully support integrated KMC-HBNC programs. Future discussions should focus on policy inclusion, resource allocation, workforce strengthening, training and community engagement strategies to support scalable and sustainable KMC programs.

## Data Availability

The original contributions presented in the study are included in the article/Supplementary Material, further inquiries can be directed to the corresponding author.
